# Outcomes of Incisional Hernia Repair Surgery After Multiple Re-recurrences: A Propensity Score Matched Analysis

**DOI:** 10.1007/s00268-021-05952-5

**Published:** 2021-01-31

**Authors:** Dimitri Sneiders, Gijs H. J. de Smet, Floris den Hartog, Yagmur Yurtkap, Anand G. Menon, Johannes Jeekel, Gert-Jan Kleinrensink, Johan F. Lange, Jean-François Gillion

**Affiliations:** 1 Department of Surgery, Erasmus University Medical Center, Rotterdam, The Netherlands; 2 Department of Surgery, IJsselland Ziekenhuis, Capelle aan den IJssel, The Netherlands; 3 Department of Neuroscience-Anatomy, Erasmus University Medical Center, Rotterdam, The Netherlands; 4 Unité de Chirurgie Viscérale Et Digestive, Hôpital Privé D’Antony, Antony, France

## Abstract

**Background:**

Patients with a re-recurrent hernia may account for up to 20% of all incisional hernia (IH) patients. IH repair in this population may be complex due to an altered anatomical and biological situation as a result of previous procedures and outcomes of IH repair in this population have not been thoroughly assessed. This study aims to assess outcomes of IH repair by dedicated hernia surgeons in patients who have already had two or more re-recurrences.

**Methods:**

A propensity score matched analysis was performed using a registry-based, prospective cohort. Patients who underwent IH repair after ≥ 2 re-recurrences operated between 2011 and 2018 and who fulfilled 1 year follow-up visit were included. Patients with similar follow-up who underwent primary IH repair were propensity score matched (1:3) and served as control group. Patient baseline characteristics, surgical and functional outcomes were analyzed and compared between both groups.

**Results:**

Seventy-three patients operated on after ≥ 2 IH re-recurrences were matched to 219 patients undergoing primary IH repair. After propensity score matching, no significant differences in patient baseline characteristics were present between groups. The incidence of re-recurrence was similar between groups (≥ 2 re-recurrences: 25% *versus* control 24%, p = 0.811). The incidence of complications, as well as long-term pain, was similar between both groups.

**Conclusion:**

IH repair in patients who have experienced multiple re-recurrences results in outcomes comparable to patients operated for a primary IH with a similar risk profile. Further surgery in patients who have already experienced multiple hernia re-recurrences is justifiable when performed by a dedicated hernia surgeon.

**Supplementary Information:**

The online version of this article (10.1007/s00268-021-05952-5) contains supplementary material, which is available to authorized users.

## Introduction

Incisional hernia (IH) remains a frequent complication after open abdominal surgery [1]. The results of IH repair have improved due to standardized use of a mesh, nevertheless, re-recurrence rates remain high, up to 20% [2, 3]. The overall risk of re-recurrence after ventral and IH repair after a median follow-up time of 41 months is estimated between 8 and 37% [4].

When left untreated, IH may cause pain or discomfort, as well as aesthetic complaints, which negatively affect quality of life [5]. Additionally, in rare cases (4–15%) IH may incarcerate [6]. Incarceration is associated with increased morbidity and mortality and requires emergency surgery [7].

Although techniques and results of abdominal wall reconstruction are improving, hernia surgeons continue to be faced with patients presenting after multiple IH re-recurrences [2]. Repair of IH after multiple re-recurrences can be technically challenging due to previous use of different techniques, which may result in damaged anatomical planes and altered tissue quality, and therefore more limited reconstructive options leading to unfavorable surgical outcomes. Outcomes after IH repair have been studied thoroughly. However, outcomes and utility of IH repair in patients who present after two or more re-recurrences remain unknown.

This study aims to assess outcomes of IH repair by dedicated hernia surgeons in patients who have had two or more previous re-recurrences. Outcomes of repair in this population will be assessed, considering postoperative complications, relief of preoperative symptoms, and IH re-recurrence.

## Materials and methods

This prospective cohort study was conducted following the STROCSS, STROBE statements, and the recommendations of the European Registry of Abdominal Wall Hernias (EuraHS) [8–10].

### Study design

A prospective, registry-based study was conducted. Adult patients who underwent IH repair after two or more re-recurrences operated between 2011 and 2018 were selected from the French Hernia-Club registry. Only patients who fulfilled their 1-year follow-up visit were included. Subsequently, control patients with similar follow-up who underwent primary IH repair were selected. IH re-recurrence was defined as: ‘A protrusion of the contents of the abdominal cavity or preperitoneal fat through a defect in the abdominal wall at the site of a previous repair of an abdominal wall hernia’ as described by Muysoms et al*.*[10]. Patients operated after two or more IH re-recurrences were compared to a 1:3 propensity score matched control group.

### Hernia-club registry

This study was executed conducted within the French Hernia-Club registry, which is a collaborative, prospective, anonymized online database of all surgical procedures for abdominal wall hernias. The French Hernia-Club registry complies to the General Data Protection Regulation and is approved by the French ‘Commission Nationale de l’Informatique et des Libertés’ (CNIL registration number: 1993959v0). Because this study is registry-based and guarantees completely anonymized data, additional participant approval and consent was not required according to the French and Dutch national ethical standards.

Surgeons specialized in abdominal wall surgery performed all operations. Each dedicated hernia surgeon must perform at least 100 inguinal and 50 ventral hernia repairs annually. Furthermore, each surgeon must accept and sign the Charter of Quality, which states that: ‘all input must be registered in a consecutive, unselected and exhaustive manner and in real time.’ A total of 191 parameters were collected by the operating surgeon and the blinded, independent, clinical research associates, using online forms. Parameters comprise data from screening, pre-, peri-, and postoperative periods. Participants consent to random peer review of original medical charts to ensure high-quality data. The medical records were checked in the case of any discrepancies. All collected parameters in this database were fully compatible with the EuraHS international online platform and the European Hernia Society (EHS) classification of abdominal wall hernias [10, 11].

### Data extraction

Relevant baseline, surgical, and functional outcome variables were extracted from the Hernia-Club database. Extracted baseline variables comprised: age, sex, body mass index (BMI), American Society of Anesthesiology (ASA) classification, diabetes mellitus, number of previous hernia re-recurrences, smoking status, IH location (medial *vs.* lateral), EHS width classification, mesh location (IPOM, sublay, onlay, no mesh), emergency surgery, synchronous repair of multiple defects, wound classification (clean, clean-contaminated, contaminated, dirty), and follow-up time. Surgical outcomes comprised: IH re-recurrence, IH repair surgery, radiological re-recurrence only, postoperative complications (surgical and medical) and Clavien–Dindo classification grade. Functional outcomes were assessed with a follow-up survey after approximately one-year post-surgery. Extracted data of this survey comprised the sensation of a non-solid scar, subjective presence of bulging, presence of pain or discomfort, and presence of daily life limitations related to the IH repair (no limitations, some limitations, or severe limitations).

### Statistical analysis

Statistical analysis was performed with R-studio (R-version: 4.0, © 2009–2020 RStudio). Discrete variables were presented as absolute numbers with percentages. Continuous variables were presented as median and interquartile range. Discrete variables were statistically compared with the chi-square test and continuous variables were compared either with the Student-T test or Mann–Whitney U test as appropriate (*i.e.*, normality was assessed graphically in quantile–quantile plot). Two groups were defined: the study group consisting of patients operated on IH after two or more re-recurrences and the control group who underwent primary IH repair. The proportion of missing data was assessed and is presented in the Online Resource 1 for each variable. Missing data were primarily caused by missing data entries and were assumed to be mostly missing at random (*i.e.*, not related to the outcomes or study groups). Multiple imputations were performed to allow the use of all available data. The following variables were included in the imputation model: age, sex, BMI, ASA-class, hernia location, EHS width classification, mesh location, emergency surgery, synchronous repair of multiple defects, diabetes mellitus, wound class, smoking status, previous hernia re-recurrence, IH re-recurrence at 1-year follow-up (predictor only), and any complications (predictor only). Continuous variables were imputed according to the predictive mean matching method, discrete variables with the use of logistic regression. In total, ten imputations for each missing value were performed. Propensity scores were calculated for each imputed dataset. The following variables were included in the propensity score model: age, sex, BMI, ASA classification, hernia location, EHS width classification, mesh location, emergency surgery, synchronous repair of multiple defects, diabetes mellitus, wound class, and smoking status. The propensity scores of the imputed datasets were pooled. Subsequently, cases (patients operated after two or more previous re-recurrences) were matched 1:3 to control patients. Cases were matched according to the nearest neighbor method, matching from the highest to lowest propensity scores. Cases and controls with propensity scores outside the region of common support were discarded. For all variables included in the propensity model, the performance of the matching model was assessed visually by plotting the mean of each covariate against the propensity score (Online Resource 2, 3, and 4). Additionally, the balance was assessed with the use of the chi-square, Student-T test or Mann–Whitney U test as appropriate. Finally, the outcomes were assessed with simple univariable analysis in the raw and matched study sample. A p-value below 0.05 was considered statistically significant.

## Results

### Patient characteristics

Seventy-six patients operated after two or more IH re-recurrences were available in the Hernia-Club registry dataset, and 763 control patients who underwent primary IH repair with similar follow-up data were available (Table 1). In the raw, unmatched sample significant differences were present between the control and study groups. BMI was higher among the re-recurrence group (control: 27.8 kg/m^2 *vs.* ≥ 2 re-recurrences: 31 kg/m^2, *p* < 0.001). Slightly more patients were operated for a laterally (around *linea semilunaris*) located IH in the control group (control: *n* = 129, 18% *vs.* ≥ 2 re-recurrences *n* = 6, 8%, *p* = 0.010) and slightly more for a combined lateral and medial hernia among the re-recurrence group (control: *n* = 45, 6% *vs.* ≥ 2 re-recurrences: *n* = 10, 13%, *p* = 0.01). Patients in the re-recurrence group presented with larger IHs more often (control: EHSII *n* = 249, 34%; EHSIII *n* = 5, 15% *vs.* ≥ 2 re-recurrences: EHSII *n* = 34, 47%, EHSIII *n* = 18, 25%, *p* < 0.001). More patients in the control group had received mesh repair with IPOM mesh reinforcement, whereas more patients in the re-recurrence group had received no new mesh reinforcement (control: IPOM: *n* = 299, 40%; no mesh *n* = 66, 9% *vs.* ≥ 2 re-recurrences: IPOM: *n* = 24, 32%, no mesh: *n* = 12, 16%, p = 0.010). The prevalence of a contaminated surgical site was higher in the re-recurrence group compared to the control group (control: *n* = 73, 10% *vs.* ≥ 2 re-recurrences: *n* = 14, 18%, *p* = 0.002). After propensity score matching, adequate balance between the control group (*n* = 219) and re-recurrence group (*n* = 73) was obtained on all covariates (Online Resource 3), and no more significant differences were present on baseline covariates between the two groups (Table 1). Three patients in the re-recurrence group could not be matched to a control patient, since the propensity scores were outside the region of common support (Online Resource 2).

**Table 1:** Patient characteristics

	Unmatched sample			Propensity score matched sample		
	control	≥ 2 re-recurrences	P	control	≥ 2 re-recurrences	P
**N**	763	76		219	73	
**Sex (male)**	378 (50)	33 (43)	0.309	94 (43)	32 (44)	0.891
**Age**	66 (56–74)	66 (57–71)	0.392	64 (55–73)	66 (58–71)	0.854
**BMI**	28 (25–32)	31 (28–34)	** < 0.001**	29 (26–35)	31 (28–34)	0.079
**ASA**						
*I-II*	561 (74)	52 (68)	0.266	152 (69)	50 (68)	0.911
*III-IV*	194 (26)	24 (32)		64 (29)	23 (32)	
**Diabetes mellitus**	133 (18)	19 (25)	0.108	55 (26)	18 (25)	0.874
**Number of previous re-recurrences**	NA			NA		
*Second re-recurrence*	NA	47 (62)		NA	45 (62)	
*Three or more re-recurrences*	NA	29 (38)		NA	28 (38)	
**Smoking**						
*Never smoked*	406 (59)	35 (52)	0.279	102 (51)	34 (53)	0.972
*Former smoker* > *1 year*	158 (23)	22 (33)		63 (32)	20 (31)	
*Incidental smoker*	25 (4)	1 (2)		5 (3)	1 (2)	
*Daily smoker*	100 (15)	9 (13)		29 (15)	9 (14)	
**Hernia location**						
*Medial*	560 (76)	59 (79)	**0.010**	168 (79)	58 (81)	0.887
*Lateral*	129 (18)	6 (8)		16 (8)	6 (8)	
*Medial and lateral*	45 (6)	10 (13)		28 (13)	8 (11)	
**EHS width classification**						
< *4 cm*	372 (51)	21 (28)	** < 0.001**	61 (29)	21 (30)	0.927
*4–10 cm*	249 (34)	34 (47)		102 (48)	32 (46)	
> *10 cm*	105 (15)	18 (25)		48 (23)	17 (24)	
**Mesh location**						
*IPOM*	299 (40)	24 (32)	**0.010**	65 (30)	24 (33)	0.912
*Sublay*	372 (49)	35 (47)		111 (52)	35 (49)	
*Onlay*	16 (2)	4 (5)		7 (3)	2 (3)	
*No mesh*	66 (9)	12 (16)		32 (15)	11 (15)	
**Emergency surgery**	29 (4)	6 (8)	0.090	19 (9)	5 (7)	0.622
**Synchronous repair of multiple defects**	110 (15)	9 (12)	0.567	32 (15)	9 (13)	0.655
**Wound classification**						
*Clean*	685 (90)	62 (82)	**0.002**	183 (84)	60 (85)	0.981
*Clean contaminated*	45 (6)	10 (13)		29 (13)	10 (14)	
*Contaminated*	23 (3)	1 (1)		2 (1)	1 (1)	
*Dirty*	5 (1)	3 (4)		5 (2)	2 (3)	
**Follow-up (years)**	2 (1–2)	1 (1–2)	0.632	1 (1–2)	1 (1–2)	0.923

*Continuous variables are presented as median and interquartile range, and discrete variables are presented as absolute number and percentage. BMI: body mass index, ASA: American society of anesthesiology, EHS: European hernia society, IPOM: intraperitoneal onlay mesh, NA: not applicable*

### Surgical outcomes

After a median follow-up of 12.4 months, 121 (16%) control patients and 18 (25%) patients operated after two or more re-recurrences had been diagnosed with a re-recurrent IH in the unmatched sample (*p* = 0.04, Table 2). In the propensity score matched sample, the re-recurrence rates among the re-recurrence group and control group were equal (control: *n* = 51, 23% *vs.* ≥ 2 re-recurrences: *n* = 18, 25%, *p* = 0.811, Table 2). Postoperative complications occurred less frequently among the re-recurrence group (*n* = 9, 13%) as compared to the control group (*n* = 44, 21%) (*p* = 0.123). Additionally, no complications among the re-recurrence group were of higher severity according to the Clavien–Dindo classification [12] as compared to the control group.

**Table 2:** Surgical outcomes

	Unmatched sample			Propensity score matched sample		
	Control	≥ 2 re-recurrences	P	Control	≥ 2 re-recurrences	P
**N**	763	76		219	73	
**IH re-recurrence**	121 (16)	19 (25)	**0.04**	51 (23)	18 (25)	0.811
*Operated re-recurrence*	28 (4)	4 (5)	0.121	14 (6)	4 (6)	
*Purely radiological re-recurrence*	25 (3)	4 (6)	0.361	11 (5)	4 (6)	
**Any postoperative complication (30 days)**	173 (23)	10 (14)	0.065	44 (21)	9 (13)	0.123
Clavien–Dindo score* (n)	744	73		197	64	
*I-IIIa*	130 (18)	10 (14)	0.175	24 (11)	8 (13)	0.61
*IIIb*	9 (1)	0		5 (3)	0	
≥ *IV*	29 (4)	0		3 (2)	0	
Type of complication* (n)	740	71		210	71	
* Surgical complication*	122 (17)	6 (9)	0.076	36 (17)	6 (8.5)	0.081
* Medical complication*	69 (9)	3 (4)	0.138	13 (6)	2 (3)	0.274

Outcomes are presented as absolute numbers and percentage. * Outcome was not available in all patients. IH: incisional hernia

### Functional outcomes

A total of 696 included patients had filled in a follow-up survey after approximately one year. In the unmatched sample, more patients in the re-recurrence group reported frequent complaints of moderate to severe pain (control: *n* = 40, 6% *vs.* ≥ 2 re-recurrences: *n* = 10, 16% *p* = 0.043, Table 3). However, in the propensity score matched sample the prevalence of moderate to severe pain was not significantly different among both groups (control: *n* = 14, 8% *vs.* ≥ 2 re-recurrences: *n* = 8, 15% *p* = 0.59, Table 3). The prevalence of bulging and the sensation of a non-solid scar was equal among both groups (Table 3). No significant difference was present in the number of patients who experienced limitations in daily life as a result of a re-recurrent IH or repair procedure.

**Table 3:** Functional outcomes

	Unmatched sample			Propensity score matched sample		
	control	≥ 2 re-recurrences	P	control	≥ 2 re-recurrences	P
**N**	634	62		172	59	
**Sensation of non-solid scar**	69 (11)	11 (18)	0.102	24 (14)	10 (17)	0.51
**Bulging**	92 (14)	13 (21)	0.164	29 (16)	12 (20)	0.38
**Any pain or discomfort**	147 (23)	20 (32)	0.097	44 (24)	18 (31)	0.344
* Moderate pain VAS: 3–6*	33 (5)	7 (11)	**0.046**	11 (6)	7 (12)	0.143
* Severe pain VAS* ≥ *6*	7 (1)	3 (5)	**0.017**	3 (2)	2 (3)	0.419
Limitation of general activities* (n)	631	62		179	59	
* Moderate limitations*	37 (6)	3 (5)	0.484	11 (6)	3 (5)	0.803
* Severe limitations*	22 (4)	4 (6)		6 (3)	3 (5)	

Outcomes are presented as absolute numbers and percentage. * Outcome was not available in all patients. VAS: visual analogue scale. Moderate limitations: difficulties in several daily activities. Severe limitations: some daily activities not possible

## Discussion

This study explored the outcomes of IH repair in patients who have had multiple (≥ 2) previous re-recurrences. The one-year prevalence of IH re-recurrence in this population of patients was relatively high (25%). However, outcomes of these patients appeared very similar to control patients with a similar risk profile. Moreover, the rate of severe complications was not higher in the studied population with multiple previous hernia re-recurrences as compared to the control patients. Additionally, adequate functional outcomes with reference to complaints of bulging, discomfort, and daily life limitations were obtained, which were again very similar to control patients.

To our knowledge, no previous data on the currently studied population of patients, with multiple previous re-recurrences of IH, is available in literature. However, a hernia re-recurrence is generally considered an important risk factor for an unfavorable outcome after IH repair, both with reference to complications and risk for re-recurrence [7]. In particular, patients with multiple previous re-recurrences are considered as complex IH patients given the fact that anatomical planes and tissue quality may have been altered by previous repair surgery, which may limit reconstructive options. These assumptions can influence the operating surgeon and the patient to refrain from a third or even fourth hernia repair procedure.

Nevertheless, based on current data, the outcomes after IH repair in patients who were operated after multiple re-recurrences appear similar to those patients with a comparable risk profile operated primarily. Patients experienced little postoperative complications and functional outcomes were comparable to previous reports [13–16]. Therefore, when performed by a dedicated hernia surgeon, a third or fourth hernia repair appears far from a futile procedure. Consequently, the operating surgeon should not directly refrain from operating upon a patient with multiple previous hernia re-recurrences and consult a dedicated hernia surgeon, if present, who may operate the re-recurrence. The results of this study also encourage for centralized treatment of complex IHs and possibly reflect similarly to other subspecialties who frequently encounter and operate patients with multiple previous hernia re-recurrences.

When interpreting results of this study, the clinical workup of patients must be taken into account. It is likely that only a minority of patients with multiple re-recurrences undergo further surgery, either due to comorbidity, technical concerns, or the wish of the patient. Currently, the relatively favorable results will in part reflect the clinical decision-making process of operating surgeons. However, the results in matched control patients undergoing a first IH repair appear similar. Therefore, we may argue that an IH re-recurrence by itself is not an important independent risk factor for an unfavorable outcome after hernia repair. Rather, the patient population with multiple IH re-recurrences consists, by definition, of patients with an unfavorable risk profile resulting in these observations.

Based on a previous inquiry by Helgstrand et al*.*[4] on reasons not to undergo further surgery after IH re-recurrence main reported reasons were the absence of symptoms (58%) and recommendations against surgery by the general practitioner or surgeon (34%) (*n* = 56). However, in symptomatic patients who are reasonably fit for surgery one may still expect a satisfactory result in the majority of patients and further surgery may very well be justifiable.

It remains unclear why some patients will develop multiple IH re-recurrences despite many improvements in surgical technique, mesh reinforcement, and specialization of surgeons. As reflected by the unmatched baseline characteristics, patients with multiple re-recurrences usually have an unfavorable risk profile. The latter group presents with higher rates of comorbidity, higher BMI, larger defects, and surgical site related (wound) problems. However, likely other, less frequently recorded factors, may play a role. Collagen remodeling, for example, may not be equally sufficient in all patients [17–20].

Based on current and available published data, no recommendation can be made on the preferable technique for re-recurrent IH repair. It has been previously suggested to use laparoscopic methods when possible [2]. The technique used may be dependent on the quality of available anatomical planes, possibly disturbed by in situ meshes and not every technique may be possible in every patient. Usually, it is preferred to use a different anatomical plane compared to the previous procedure. In case of large defects, additional component separation techniques, if not already performed, could be considered to ensure tension-free closure [21–23].

### Limitations

The presented data are observational and the results may be influenced by pre-inclusion selection bias and reflect good clinical decision-making by the participating surgeons. Although the matched study design assured to an extent compatibility of groups, pre-inclusion selection bias likely occurred and may cause exclusion of patients with an unfavorable risk profile. Since all data are observational, causality of associations found cannot be confirmed and should be interpreted with caution. Due to the limited available data and the matched study design, a multivariable approach to identify risk factors for an unfavorable outcome is not possible. However, given the more extensive clinical workup present in re-recurrent IH patients, estimates obtained in multivariable models will inherently be influenced by selection bias. Therefore, to study outcomes, a matched approach is probably preferred. Propensity score matching will help to balance covariates across groups, but will not necessarily result in individually matched pairs. Therefore, on a per case basis differences could still be present between the studied populations. Also, unknown risk factors, or factors not included in the propensity score model may still be unbalanced.

## Conclusions

IH repair surgery in patients who experienced multiple (≥ 2) re-recurrences results in outcomes comparable to patients operated on a first IH with a similar risk profile. Re-recurrence rates were 24.7% in the group of patients who experienced multiple re-recurrences and 23.3% in in comparable controls undergoing their first IH repair. If performed by a dedicated hernia surgeon, further surgery in patients who already experienced multiple IH re-recurrences results in good outcomes and is a justifiable treatment.

## Supplementary Information

Below is the link to the electronic supplementary material.

Supplementary Information1 (DOCX 15 kb)

Supplementary Information1 (TIFF 20 kb)

Supplementary Information1 (TIFF 762 kb)

Supplementary Information1 (TIFF 255 kb)
