# Supplementary material for: Outcomes of Incisional Hernia Repair Surgery After Multiple Re-recurrences: A Propensity Score Matched Analysis
Source: World J Surg. 2021 Jan 31;45(5):1425–32. doi: 10.1007/s00268-021-05952-5 (PMC8026468; doi:10.1007/s00268-021-05952-5)
Supplement: Supplementary file 1 — Supplementary Information1 (DOCX 15 kb) [file 268_2021_5952_MOESM1_ESM.docx]

**Supplemental Table.** Overview of missing data

|  | **Unadjusted sample** | **Propensity score matched sample** |
| --- | --- | --- |
| **N** | 839 | 292 |
| **Sex (male)** | 0 | 0 |
| **Age** | 3 (0.4) | 3 (1) |
| **BMI** | 18 (2.1) | 7 (2.4) |
| **ASA** | 9 (1.1) | 3 (1) |
| **Diabetes mellitus** | 17 (2) | 8 (2.7) |
| **Number of previous recurrences** | 0 | 0 |
| **Smoking** | 93 (11.1) | 29 (9.9) |
| **Hernia location** | 33 (3.9) | 8 (2.7) |
| **EHS width classification** | 42 (5) | 11 (3.8) |
| **Mesh location** | 11 (1.3) | 5 (1.7) |
| **Emergency surgery** | 5 (0.6) | 0 |
| **Wound classification** | 5 (0.6) | 7 (2.4) |
| **Follow-up (years)** | 57 (6.8) | 15 (5.1) |

*Missing data are presented as absolute numbers and percentage. BMI: body mass index, ASA: American society of anesthesiology, EHS: European hernia society.*
