# Supplementary figures and images for: Outcomes of Incisional Hernia Repair Surgery After Multiple Re-recurrences: A Propensity Score Matched Analysis
Source: World J Surg. 2021 Jan 31;45(5):1425–32. doi: 10.1007/s00268-021-05952-5 (PMC8026468; doi:10.1007/s00268-021-05952-5)

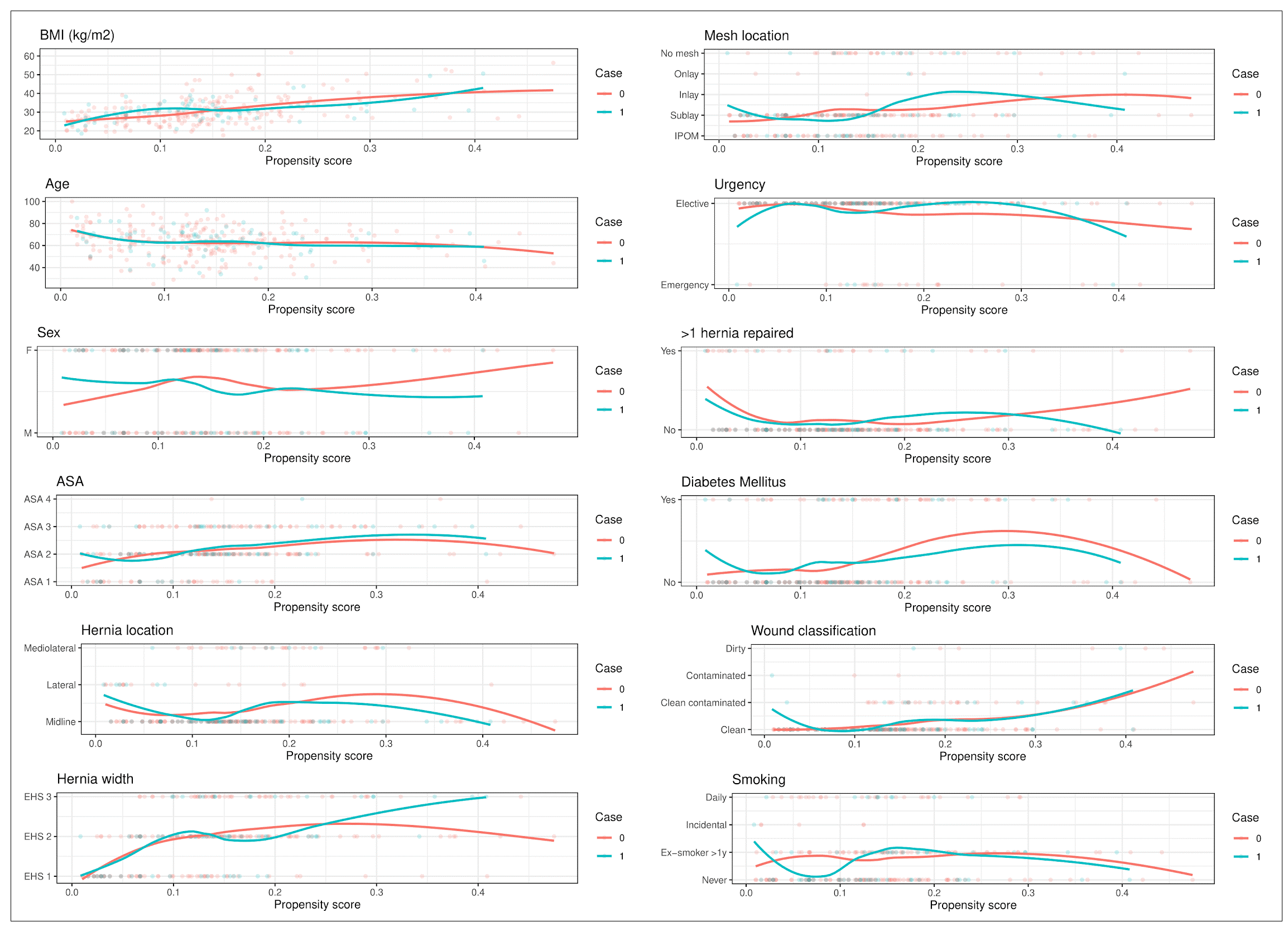

Supplement: Supplementary file 3 — Supplementary Information1 (TIFF 762 kb) [file 268_2021_5952_MOESM3_ESM.tiff]

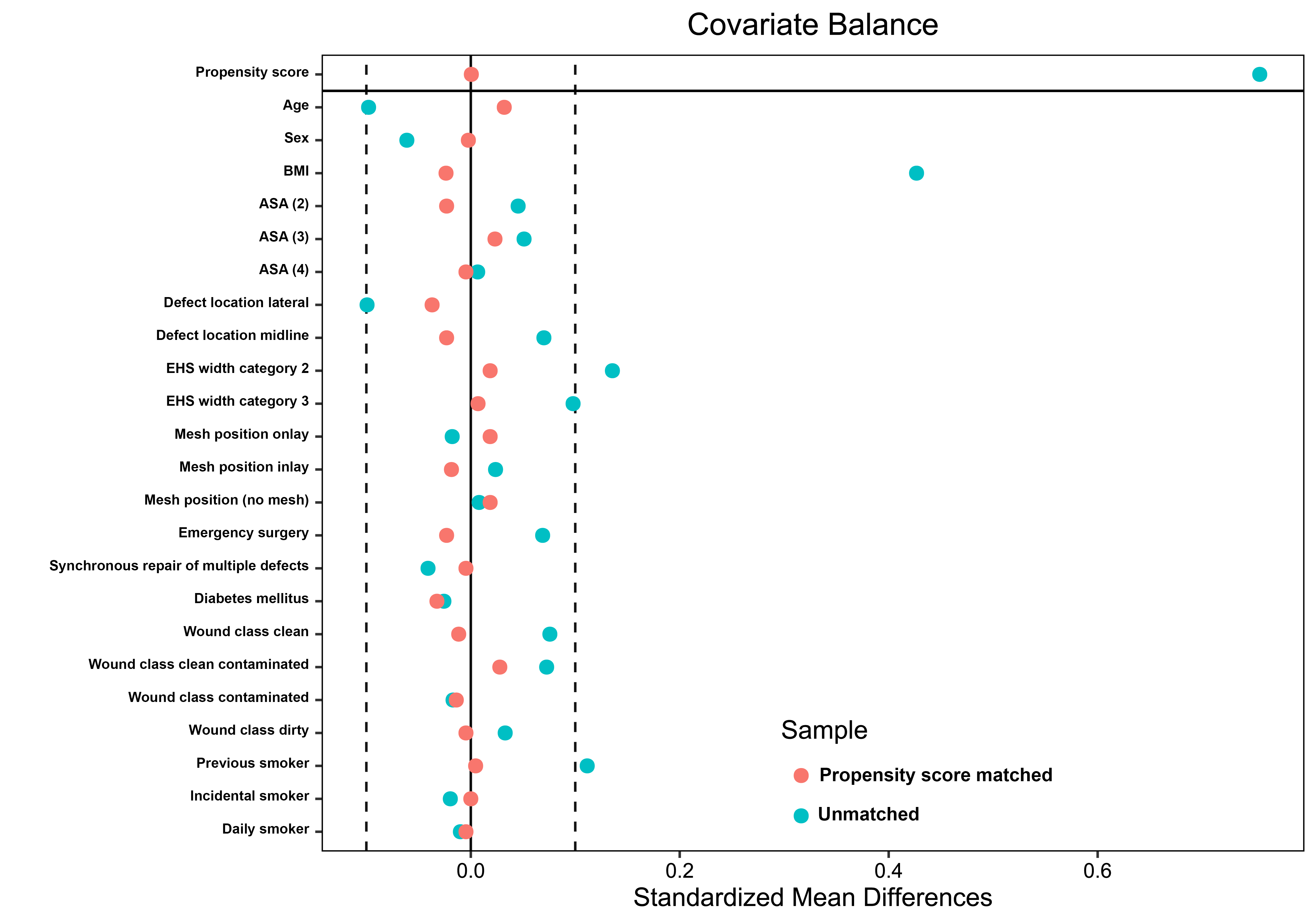

Supplement: Supplementary file 4 — Supplementary Information1 (TIFF 255 kb) [file 268_2021_5952_MOESM4_ESM.tiff]
